# Contributions of Ocular Surface Components to Matrix-Metalloproteinases (MMP)-2 and MMP-9 in Feline Tears following Corneal Epithelial Wounding

**DOI:** 10.1371/journal.pone.0071948

**Published:** 2013-08-19

**Authors:** Andrea Petznick, Michele C. Madigan, Qian Garrett, Deborah F. Sweeney, Margaret D. M. Evans

**Affiliations:** 1 Vision CRC, Sydney, Australia; 2 School of Optometry and Vision Science, University of New South Wales, Sydney, Australia; 3 Save Sight Institute, University of Sydney, Sydney, Australia; 4 Brien Holden Vision Institute, Sydney, Australia; 5 University of Western Sydney, Penrith, Australia; 6 CSIRO Materials Science and Engineering, Sydney, Australia; Medical College of Georgia, United States of America

## Abstract

**Purpose:**

This study investigated ocular surface components that contribute to matrix-metalloproteinase (MMP)-2 and MMP-9 found in tears following corneal epithelial wounding.

**Methods:**

Laboratory short-haired cats underwent corneal epithelial debridement in one randomly chosen eye (n = 18). Eye-flush tears were collected at baseline and during various healing stages. Procedural control eyes (identical experimental protocol as wounded eyes except for wounding, n = 5) served as controls for tear analysis. MMP activity was analyzed in tears using gelatin zymography. MMP staining patterns were evaluated in ocular tissues using immunohistochemistry and used to determine MMP expression sites responsible for tear-derived MMPs.

**Results:**

The proMMP-2 and proMMP-9 activity in tears was highest in wounded and procedural control eyes during epithelial migration (8 to 36 hours post-wounding). Wounded eyes showed significantly higher proMMP-9 in tears only during and after epithelial restratification (day 3 to 4 and day 7 to 28 post-wounding, respectively) as compared to procedural controls (*p*<0.05). Tears from wounded and procedural control eyes showed no statistical differences for pro-MMP-2 and MMP-9 (*p*>0.05). Immunohistochemistry showed increased MMP-2 and MMP-9 expression in the cornea during epithelial migration and wound closure. The conjunctival epithelium exhibited highest levels of both MMPs during wound closure, while MMP-9 expression was reduced in conjunctival goblet cells during corneal epithelial migration followed by complete absence of the cells during wound closure. The immunostaining for both MMPs was elevated in the lacrimal gland during corneal healing, with little/no change in the meibomian glands. Conjunctival-associated lymphoid tissue (CALT) showed weak MMP-2 and intense MMP-9 staining.

**Conclusions:**

Following wounding, migrating corneal epithelium contributed little to the observed MMP levels in tears. The major sources assessed in the present study for tear-derived MMP-2 and MMP-9 following corneal wounding are the lacrimal gland and CALT. Other sources included stromal keratocytes and conjunctiva with goblet cells.

## Introduction

The ocular surface is a complex unit comprised of various epithelial and glandular tissues (cornea, bulbar and palpebral conjunctiva, lacrimal glands and accessory eyelid glands) that secrete essential tear film components. These tissues are connected by a continuous epithelium as well as the nervous, immune and endocrine systems [1], [2].

The ocular surface system is bathed by the extracellular tear film which reflects ocular and systemic health. Tears can be collected using absorbent filter paper, sponges or glass microcapillary tubes [3]. Changes in the protein composition of collected tears have been associated with ocular wound healing [4]–[6] and ocular surface conditions such as dry eye [7]–[9], meibomian gland dysfunction and keratoconjunctivitis sicca [9], [10], as well as systemic conditions including diabetes [11] and breast cancer [12].

Matrix-metalloproteinases (MMPs), specifically MMP-2 (gelatinase A) and MMP-9 (gelatinase B), are in tears and also in corneal tissue during wound healing and in ocular surface disease, including dry eye and keratoconus [4], [13]–[18]. MMPs are key effectors and regulators of inflammation, wound healing, tissue remodeling and pathogenic processes [19], [20]. Most MMPs are secreted in an enzymatically inactive state, as pro-enzymes, which are activated in a proteolytic manner by direct cleavage of the pro-peptide by another MMP or a non-proteolytic manner by organomercurials.

It is well understood that each of the ocular surface tissues contribute to the tear film makeup, thus it is more appropriate to consider the entire ocular surface microenvironment when analyzing tear proteins present during wound healing or in disease conditions [1]. However, for both anatomical and ethical reasons, tissue samples are not routinely collected from patients in clinical research. Hence, there has been no thorough investigation of which ocular surface tissues secrete MMP-2 and/or MMP-9 into the tear film during corneal wound healing. The current study aimed to address this issue by identifying ocular surface tissues that contribute to the MMP levels detected in tears following a corneal epithelial debridement in a feline model.

## Materials and Methods

### Animal Model

The feline model has been chosen for the current study as it has been successfully used for wound healing studies in refractive surgery [21]–[23] and following corneal epithelial removal using heptan-1-ol [24], investigations of corneal sensitivity and nerve regeneration after non-penetrating keratotomy or penetrating autografts [25] and analysis of tear proteins following corneal epithelial debridement [26]. Different to most animal models, the feline cornea possesses fibrils that resemble a Bowman’s membrane [27], [28] and a similar corneal epithelial thickness as a human [29], [30].

Furthermore, analysis of MMP levels following a corneal epithelial debridement wound in a feline model has been previously published [26]. Specifically, the study showed that proMMP-9 levels in feline tears were highest on day 1 and reduced to lower levels by day 3.

### Experimental Animals

Studies were conducted in accordance with the ARVO Statement for the Use of Animals in Ophthalmic and Vision Research and with approval from the Vision CRC and Brien Holden Vision Institute Animal Ethics Committee (Permit number: VIAC 06/04). Twenty-three laboratory adult short-haired cats (14 males, 13 females; 1.8±0.9 years; 4.1±1.1 kg) were included. Consistent with practice in the host institution, nictitating membranes were removed (as previously described [31]) from both eyes of each animal at least 8 weeks prior to enrolment in the study. At the time of enrolment, no clinical signs of ocular surface disturbance were observed using a slit lamp.

### Experimental Design

Surgical procedures on animals were performed under general anesthesia: intramuscular injection of ketamine 3 mg/kg body weight, acepromazine maleate 0.05 mg/kg body weight (Delvet, Seven Hills, Australia) and atropine sulphate 0.02–0.04 mg/kg body weight (Apex Laboratories, Somersby, Australia), and 5% isoflurane vaporized in 2 L oxygen (Veterinary Companies of Australia, Kings Park, Australia) delivered through an endotracheal tube. Upon anesthesia, the analgesic, buprenorphine hydrochloride 0.005–0.01 mg/kg (Reckitt Benckiser Healthcare Limited, Hull, UK), was administered subcutaneously. Eye drops were administered into the randomly selected eye (hereafter referred to as the experimental eye) prior to the procedure as follows: local anaesthetic, 0.4% oxybuprocaine hydrochloride; ophthalmic antibiotic, 0.3% tobramycin; ophthalmic cycoplegic, 0.5% tropicamide (Bausch & Lomb, Sydney, Australia). The eyelids of the experimental eye were retracted with a Barraquer wire lid speculum and rinsed with sterile saline. The globe was maintained in a central position using forceps.

A central corneal epithelial debridement 9 mm in diameter was created in the experimental eye of each animal by the same surgeon (AP) according to a surgical procedure previously reported [26]. Prior to performing this debridement procedure on cats, the surgeon practiced corneal epithelial debridement on bovine eyes sourced fresh from the local abattoir checking each debridement by routine histology to ensure the corneal epithelium was completely removed without disruption to the basement membrane or anterior stroma. After surgery, the animal was placed on a warming blanket and Hartman’s Solution (Compound sodium lactate intravenous infusion BP; Baxter Healthcare, Sydney, Australia) was administered subcutaneously. Eight hours after wounding, the analgesic buprenorphine hydrochloride (0.005–0.01 mg/kg) was given subcutaneously to alleviate any discomfort or pain to the animal.

Procedural control animals served as controls for the analysis of tears. These procedural control animals were not wounded, but received identical medications and experimental procedures as the wounded animals, including general anesthesia, topical eye drops, eyelid retraction, positioning of the eyeball using forceps and post-operative medications. Eye-flush tears were collected from both wounded and procedural control eyes after instillation of 10 µl of sterile saline and immediately placed on ice [26], [31]. Tear samples were stored at −80°C until analysis. Tears were collected at the baseline prior wounding and at various time points post-wounding (8, 16, 24 and 36 hours post-wounding; 48 hours post-wounding and wound closure; 3, 4, 5, 6, 7, 10, 14, 18, 21, 26 and 28 days post-wounding). Individual tear samples were analyzed each for the total protein content and MMP-2 and MMP-9 activity (see tear analysis). One to three animals were sacrificed with an overdose of sodium pentobarbital (Virbac Australia Pty, Sydney, Australia) at each predetermined time point (prior to wounding; 8, 16 and 24 hours post-wounding, 48 hours and wound closure; 7, 14 and 28 days post-wounding). Ocular surface tissues, specifically the corneas, conjunctivas, lacrimal glands and eyelids, were collected at each time point and fixed in 4% neutral buffered formalin, paraffin embedded and prepared for MMP-2 and MMP-9 expression (see tissue analysis).

The time points were categorized as different healing phases by daily slit lamp and post-mortem histology examination of each wounded eye. These phases are listed in Table 1 and are as follows: 1) prior to wounding (as tissue controls); 2) following wounding, during early reepithelisation (at 8 and 16 hours post-wounding); 3) late epithelial migration (at 24 and 36 hours post-wounding); 4) wound closure (43 to 50 hours post-wounding); 5) early restratification (at 3 and 4 days post-wounding); 6) late phase restratification (at 5 and 6 days post-wounding); and 7) following full restratification (at 7, 14 and 28 days post-wounding). Each of the analyzed tear samples from wounded and procedural control eyes were grouped into healing phases as outlined in Table 1 to enable a statistical comparison between various stages of wound healing.

**Table 1:** Tear sample grouping based on wound healing stages for both wounded and procedural control eyes.

Stages	Morphological characteristics ofwound healing	Pooled time points (post-wounding)	No. of samples forwounded animals	No. of samples for proceduralcontrol animals
1	**prior wounding**	0	18	5
2	**early migration**	8 and 16 hours	22	6
3	**late migration**	24 and 36 hours	16	5
4	**wound closure**	48 hours and wound closure	11	3
5	**early restratification**	3 and 4 days	12	4
6	**late restratification**	5 and 6 days	12	4
7	**fully restratified epithelium**	7, 10, 14, 18, 21, 25 and 28 days	22	8

### Tear Analysis

Total protein content from each tear sample was measured using a Pierce Micro BCA protein assay kit (Rockford, IL) following the manufacturer instructions. The MMP activity in tears was determined using gelatin zymography [26]. Briefly, a total protein content of 5 µg for each individual tear sample, a low range molecular weight standard (Precision Plus Protein Standard Kaleidoscope; Bio-Rad, Hercules, CA, USA), a gelatinase zymogram standard (dilution of 1/2000; Chemicon, Temecula, CA, USA), 1 mM 4-aminophenyl-mercuric acetate (APMA) activated MMP-9 standard (total concentration of 0.04 µg; R&D Systems) as well as 1 mM APMA activated and non-activated human fibroblast culture media were loaded into pre-cast 10% Novex zymography gelatin gels (Invitrogen, Carlsbad, CA). Separation of proteins was performed by electrophoresis at 125 Volt for 2.5 hours. Gels were rinsed in 1X renaturing buffer (Invitrogen) and incubated overnight in 1X developing buffer (Invitrogen) at 37°C for 18 hours. Gels were stained in 0.25% Coomassie Blue and destained in methanol:glacial acetic acid:distilled water (5∶1:4). The clear bands on a blue stained background indicated gelatin degradation from the presence of MMP activity. These bands were identified as pro-enzymes (pro-MMP-2 and pro-MMP-9) and activated MMP (MMP-2 and MMP-9) forms based on their molecular weight. Gels were scanned and the images were analyzed using a densitometer (GS-800, Bio-Rad, Hercules, CA) and an image analysis program (Bio-Rad Quantity one). Optical density (OD) values of clear bands were calculated and an automatic volume tool was applied to count the number of gel pixels in each band, to determine the volume of the gelatin degradation. OD values and number of gel pixels were multiplied to quantify MMP activity of each band (OD * number of gel pixels).

### Tissue Analysis

Ocular tissues collected at the different time points were fixed in 4% neutral buffered formalin for 6 hours and then transferred to 70% ethanol until processing to paraffin. Embedded tissue was routinely sectioned at 4 µm and immunostained as previously described [31]. Briefly, this involved incubation for 20 minutes in 3% H_2_O_2_ solution at room temperature, 1X universal decloaker buffer (Biocare Medical) for 20 minutes in a water bath at 80°C, primary antibody (mouse anti-human MMP-2 at 20 µg/ml or rabbit anti-human MMP-9 at 20 µg/ml detecting both pro- and activated forms, Thermo Fisher Scientific, Fremont, CA) or neat universal serum as negative control (Biocare Medical, Concord, CA) for 1 hour at room temperature. This was followed by incubation with the secondary antibody (biotin conjugated anti-mouse or anti-rabbit IgG at 30 µg/ml, Zymed Laboratories, Invitrogen, Carlsbad, CA) for 30 minutes at room temperature, streptavidin-HRP (Biocare Medical) for 30 minutes at room temperature and substrate chromogen solution (DAB chromogen system; Carpinteria, CA) for 5 minutes at room temperature. Tissue sections were extensively rinsed with 1x TRIS buffered saline (Biocare Medical) after each incubation step. Tissue sections were counterstained with haematoxylin (Fronine Pty Ltd, Sydney, Australia) and mounted in Ultramount (No.4; Fronine Pty Ltd) with coverslips. Images of sections were captured using a Nikon Digital Sight DS-5M camera with integrated software (Nikon View 6, Japan) attached to a light microscope (Zeiss Imager A1 AX10, Germany).

### Data Analysis

Differences in MMP activity between tear samples collected from wounded and procedural control eyes were statistically analyzed with a linear mixed model using the SPSS package (SPSS Statistics Gradpack 17.0; Chicago, IL, USA). A log-transformation was applied to provide variance-stability and normal data distribution. The statistical significance level was set at *p*<0.05 and a post-hoc analysis was performed to account for multiple comparisons.

## Results

### Tear Analysis

Table 2 displays activity levels of proMMP-2, proMMP-9 and MMP-9 detected in tears from wounded and procedural control eyes during each of the investigated healing phases. The statistical analysis revealed that wounded eyes had significantly increased proMMP-9, MMP-9 and proMMP-2 activity during early (8 to 16 hours) and late (24 to 36 hours) migration of the corneal epithelium (*p*<0.001, Table 2). Procedural control eyes exhibited an increase of MMP activity during the stage equivalent to early wound healing, but only proMMP-2 was found to be significantly elevated in tears collected at 8 to 16 hours and 24 to 36 hours (*p*<0.001, Table 2). A comparison between wounded and procedural control eyes showed that the wounded eyes had a significantly higher level of proMMP-9 activity during early (3 and 4 days post-wounding) and complete (7 to 28 days post-wounding) corneal epithelial restratification as compared to procedural control eyes (*p*<0.04, Table 2). There were no significant differences in the levels of pro-MMP-2 and MMP-9 between the wounded and procedural control group at any time point. Although not significant, an elevation of pro-MMP-2 levels was observed in procedural control eyes during equivalent stages of early (8 and 16 hours post-wounding) and late (24 and 36 hours post-wounding) migration of the epithelium as compared to wounded animals (*p = *0.078 and *p = *0.05 respectively). Outliers were not removed during the statistical analysis and the higher proMMP-2 activity noted in procedural control eyes at 8 and 16 hours following the procedure is due to the inclusion of the two outliers. MMP-2 was not detected in any of the tear samples (data not shown).

**Table 2:** MMP-2 and MMP-9 activity in tears.

	Time points (post-wounding)	Procedural control eyes			Wounded eyes		
		proMMP-9^1^	MMP-9^1^	proMMP-2^1^	proMMP-9^1^	MMP-9^1^	proMMP-2^1^
		Estimated mean(95% CI)	Estimated mean(95% CI)	Estimated mean(95% CI)	Estimated mean(95% CI)	Estimated mean(95% CI)	Estimated mean(95% CI)
**Prior to wounding**	0	151 (32–721)	1 (0–6)	43 (10–181)	161 (78–330)	2 (1–4)	4 (2–8)
**Early migration**	8–16 hours	488 (117–2039)	4 (1–15)	**916 (219–3790)** *	**889 (441–1772)** *	**14 (6–33)** *	**87 (43–178)** *
**Late migration**	24–36 hours	916 (206–4064)	5 (1–21)	**311 (74–1326)** *	**907 (428–1920)** *	**35 (14–88)** *	**40 (19–86)** *
**Wound closure**	48 hours, wound closure	119 (19–742)	1 (0–5)	30 (6–154)	245 (106–567)	2 (1–5)	6 (3–14)
**Early restratification**	3–4 days	**12 (2–55)** †	1 (0–4)	10 (2–46)	**81 (35–187)** †	2 (1–5)	5 (2–12)
**Late restratification**	5–6 days	18 (4–82)	1 (0–4)	7 (1–32)	106 (46–245)	2 (1–5)	6 (3–14)
**Fully restratified epithelium**	7–28 days	**86 (12–590)** †	1 (0–5)	8 (1–44)	**217 (77–608)** †	2 (1–9)	5 (2–15)

Activity of proMMP-2, proMMP-9 and MMP-9 in tears from wounded and procedural control eyes at different phases of epithelial healing.

1 Optical Density (OD) multiplied by the number of gel pixels.

* significantly greater MMP activity when compared to before wounding/procedure (p<0.001).

† significantly greater proMMP-9 activity in wounded eyes as compared to procedural control eyes (p<0.04).

### Ocular Surface Immunohistochemistry

**Figure 1:**
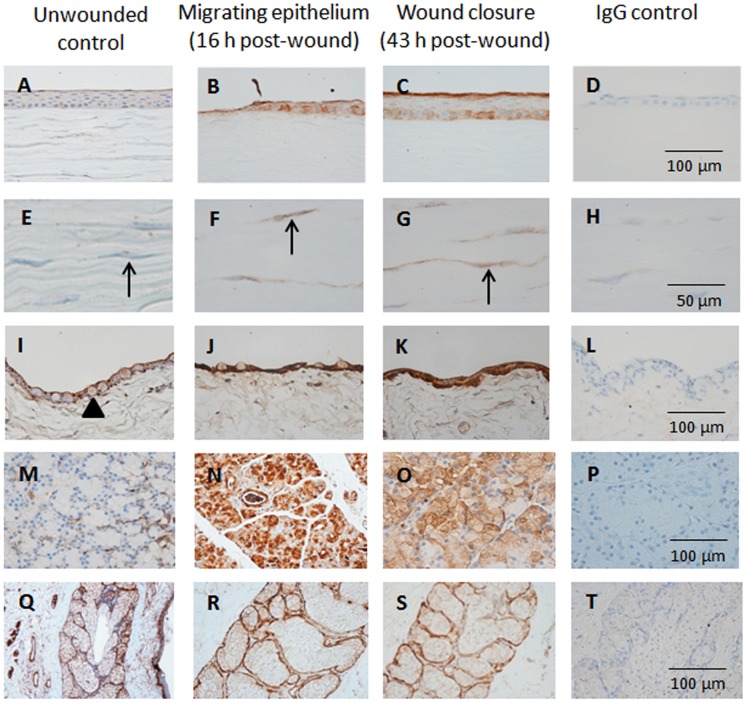
MMP-2 expression in ocular surface tissues. Representative light micrographs of tissue sections showing MMP-2 expression (A–D) corneal epithelium, (E–H) stromal keratocytes, (I–L) bulbar conjunctival epithelium, (M–P) lacrimal gland and (Q–T) meibomian gland of unwounded control eyes collected prior to surgery and wounded eyes at various phases of healing. Arrows indicate stromal keratocytes. Arrow head shows an example of a conjunctival goblet cell.

**Figure 2:**
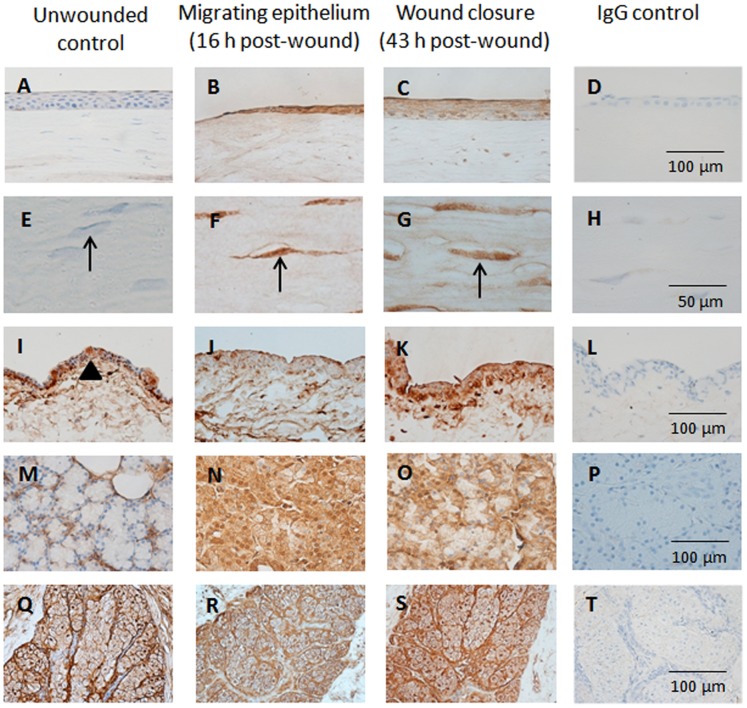
MMP-9 expression in ocular surface tissues. Representative light micrographs of tissue sections showing MMP-9 expression in (A–D) corneal epithelium, (E–H) stromal keratocytes, (I–L) bulbar conjunctival epithelium, (M–P) lacrimal gland and (Q–T) meibomian gland of unwounded control eyes collected prior to surgery, and wounded eyes at various stages of healing. Arrows indicate stromal keratocytes. Arrow head shows an example of a conjunctival goblet cell.

The immunoreactivity of MMP-2 and MMP-9 in the corneal, conjunctival, lacrimal gland and meibomian gland tissues of wounded and unwounded control eyes are depicted in Figures 1 and 2. Examination of the tissue sections stained with the different antibodies and visualized with DAB which reveals brown staining of positive antibody responses, showed little or no detectable MMP-2 and MMP-9 in the epithelium and stroma of unwounded corneas (Figures 1A, 1E, 2A and 2E). In wounded corneas, increased MMP-2 and MMP-9 reactivity was observed at the epithelial wound edge at 16 hours post-wounding and at wound closure at 43 hours post-wounding (Figures 1B, 1C, 2B and 2C). At the time of wound closure, MMP-2 was concentrated to the most superficial and basal columnar epithelial cells (Figure 1C) while MMP-9 reactivity was evenly distributed in all epithelial layers (Figure 2C). Increased MMP-2 and MMP-9 immunostaining were observed in keratocytes during epithelial migration and wound closure, with particularly high levels found for MMP-9 expression (Figures 2F and 2G). After full recovery of the epithelial cell layers following wounding, expression level and distribution of MMPs in the corneal epithelium and stroma returned to the low baseline levels, similar to those seen in the unwounded corneas.

The conjunctival epithelium of unwounded eyes showed weak MMP-2 immunostaining in all cell layers, while MMP-9 was predominantly seen in the basal epithelium and conjunctival goblet cells (Figures 1I and 1I). In wounded eyes during corneal epithelial migration (Figure 1J and 1K), the conjunctival epithelium showed increased MMP-2 with distinctly higher levels observed at the time of wound closure as compared to unwounded eyes. The highest levels of MMP-9 immunoreactivity in the conjunctival epithelium were observed during wound closure (Figures 2K). We noted an absence of mucin-producing conjunctival goblet cells in the tissue collected during corneal healing at the time of wound closure (43 to 50 hours post-wounding). Once healing was completed and the corneal epithelium restratified (from day 7 post-wounding onwards), the staining pattern of MMP-2 and MMP-9 was identical to unwounded eyes with the exception of slightly reduced MMP-9 reactivity in conjunctival goblet cells.

The lacrimal glands of unwounded animals showed low intensity of MMP-2 and MMP-9 immunoreactivity (Figures 1M and 1M). The expression of both MMPs was increased during corneal healing with immunoreactivity observed in the entire acini of the lacrimal glands (Figures 1N, 1O, 2N and 2O). When the epithelium was fully restratified (day 7 post-wounding onwards), the lacrimal gland showed similar immunoreactivity of MMP-2 and MMP-9 as seen in unwounded eyes.

MMP-2 expression was similar in meibomian glands of both unwounded and wounded eyes and was observed mainly in the basement membrane of acini (Figures 1Q–S). In contrast, MMP-9 was expressed throughout all aspects of the glandular tissue of unwounded eyes (Figure 2Q) and only very slightly increased in intensity during corneal epithelial healing (Figures 2R and 2S). In meibomian glands collected from animals with a fully restratified corneal epithelium (from day 7 post-wounding onwards), the immunoreactivity of MMP-9 appeared to be diminished in comparison to unwounded eyes.

**Figure 3:**
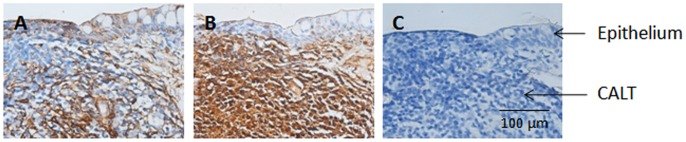
MMP-2 and MMP-9 expression in conjunctival associated lymphoid tissue (CALT) in the palpebral conjunctiva. Representative light micrographs of a section through CALT showing (A) MMP-2, (B) MMP-9 expression and (C) IgG control, at the time of corneal wound closure.

Conjunctival-associated lymphoid tissue (CALT) was detected beneath the palpebral conjunctiva in the proximity of the fornix of wounded eyes. Figure 3 presents light micrographs of MMP immunoreactivity in CALT of the palpebral conjunctiva collected at the time of wound closure. MMP-2 was mainly found between the cells comprising CALT (Figure 3A), while MMP-9 showed strong expression within the cell body and the intercellular space of CALT (Figure 3B).

## Discussion

The tear film is an integral part of the ocular surface and provides an easily accessible source of both qualitative and quantitative information to assess its health [10]. There is great interest in using diagnostic tools to detect elevated tear MMP levels to diagnose dry eye or even poor ocular surface conditions prior to surgery in an effort to improve post-surgical outcomes [32], [33]. However, it is not fully understood which of the ocular surface tissues produce the MMPs found in the tear film. The present study simultaneously explored the expression patterns of MMP-2 and MMP-9 in tears and ocular surface tissues at various stages of wound healing following corneal epithelial debridement.We observed that MMP-9 was strongly expressed within the cells at the tip of the leading edge of the migrating epithelium after wounding. When the wound had closed, MMP-9 was evenly distributed in the central epithelial area and returned to the baseline level following full restratification of the corneal epithelium (from day 7 post-wounding onwards), consistent with completion of the wound healing process. MMP-2 was also detected in corneal epithelial cells at the leading edge of migrating cells during wound healing. At the time of wound closure, MMP-2 was mainly localized at a still elevated intensity in the most outer and inner corneal epithelial cell layers and reduced to baseline levels after full recovery of the corneal epithelium (from day 7 post-wounding onwards).

MMP-9 has been recently reported to play an important role in the disassembling process of hemidesmosomes, the epithelium-basement membrane cell surface attachment sites in corneal epithelium [34]. MMP-9 was found to specifically cleave the membrane protein β4 integrin within the hemidesmosome, which in turn facilitates the movement of the corneal epithelial sheet across the basement membrane to close the wound [34]. Therefore it is not unexpected that we observed a high level of MMP-9 expression in the healing corneal epithelium.

The observed distribution pattern of MMP-2 and MMP-9 during corneal epithelial migration in the present study was similar to that observed in a mouse debridement model [35]. In contrast to the present study however, an *in vitro* study using human corneal tissue detected only little MMP-9 in the migrating epithelium, with increased levels of MMP-9 once multiple epithelial layers were established [36] In the present study we found the highest level of MMP-9 expression in the leading edge of the healing epithelium, with its expression reduced at the time of wound closure. The different outcomes observed between these two studies could be due to the different tissue species used in the studies (human *vs* animal tissues), or, more likely, result from other factors such as the debridement technique used and/or the *in vitro* nature of the study design. *In vitro* investigations cannot totally mimic wound healing responses that occur in the eye, because important ocular surface components, including inflammatory cells and corneal nerves are not present. These components are essential in contributing to healing processes and MMP expression *in vivo*. It is also possible that MMP expression may be induced during tissue preparation of the corneas for organ culture, which was demonstrated in organ cultured rabbit corneas [37]. This finding, if considered alone, suggests that the corneal epithelial cells produce MMP-2 and MMP-9 that may be released into the tear film.

In tears, we detected increased activity of MMP-2 and MMP-9 during corneal epithelial migration, which returned to baseline levels at the time of wound closure. We used tears collected from procedural control animals (undergoing the identical experimental protocol as the wounded animals except for the wounding itself) as experimental controls for tear analysis. These procedural control eyes, however, showed similar tear MMP-2 and MMP-9 levels as wounded eyes. This indicated that the actively migrating corneal epithelium contributes very little to the observed MMP levels in tears, and other ocular surface tissues must be responsible for the detected level of MMPs in tears during corneal wound healing.

The stromal keratocytes investigated in the present study expressed MMP-2 and MMP-9 in response to corneal wounding, with particularly strong expression of MMP-9 during phases of epithelial cell migration and restratification. This was consistent with findings following superficial keratectomy in rabbit corneas [37]. It is possible that some MMPs detected in the tears from the current study were produced by the stromal keratocytes located within the vicinity of the wound bed. These MMPs may have diffused through the anterior third of the stroma into the tear film until the migrating epithelium had closed the wound. We believe this contribution of the stromal keratocytes must have been relatively small because procedural control eyes with the intact epithelium showed similar MMP expressions as wounded eyes.

The immunoreactivity of the MMP-2 and MMP-9 in the current study was significantly increased in the conjunctival epithelium following wounding until the point of wound closure. This elevation in MMP expression may be associated with an increased number of inflammatory cells observed in the conjunctival stroma sub-adjacent to the conjunctival epithelium (data not shown). Invading inflammatory cells may activate epithelial cells to produce MMPs [38]. Comparison of MMP production patterns in tears and conjunctival epithelial tissue did not however match outcomes of the current study. The highest MMP activity in tears in response to corneal wounding was detected during epithelial migration, while in conjunctival tissue, the highest MMP immunoreactivity was observed later, at the time of wound closure, when tear MMP levels were already diminished. This further suggests that the conjunctival epithelium may have only a minor contribution towards MMPs detected in the tear film.

Unwounded control eyes showed MMP-9 activity in the goblet cells of the conjunctiva. To ensure that the immunoreactivity detected was not the result of omnipresent stickiness of the mucin secreted by goblet cells, we compared MMP-9 staining to that found using a lypholized universal control serum reconstituted with antibody diluent to a concentration of 10 or 20 µg/ml. No immunoreactivity in goblet cells was observed for the universal control (Figure 3). During early wound healing, we no longer observed MMP-9 in the goblet cells, which may indicate that these cells expelled their contents of mucin and MMP-9 into the tear film contributing to the increase in tear MMP-9 activity. This is further supported by the observation that at the time of wound closure, conjunctival goblet cells appeared to be absent consistent with lower tear-derived MMP-9 levels. Conjunctival goblet cells re-appeared in tissue sections, but with a much reduced intensity of MMP-9, once the corneal epithelium had fully healed. We did not assess changes in goblet cell numbers in the procedural control animals, hence we cannot ascertain their full contribution toward MMP-9 in tears.

In the lacrimal glands from animals with wounded eyes, the expression of MMP-2 and MMP-9 was elevated during corneal epithelial migration and at the time of wound closure compared to the levels in lacrimal tissue from the unwounded eyes. When the corneal epithelium was fully restratified (from day 7 post-wounding onwards), lacrimal glands showed MMP expression comparable to that found in the lacrimal glands collected from unwounded animals. At the current time we can find no published reports on the expression of MMPs in the lacrimal gland following corneal wounding. However, increased MMP-9 levels have been associated with inflamed lacrimal glands in a rabbit dry eye model [39]. In the feline model in our study, inflammatory cells were present in the lacrimal gland, although experimental procedures did not directly interfere with the lacrimal gland. Elevated levels of MMP-9 were noted in both wounded and procedural control animals. This indicated that some of the experimental procedures and/or medications caused inflammation in the lacrimal gland which subsequently induced the acini to produce and secrete MMP-9. Furthermore, the lacrimal gland has direct access to the tear film and may hence be a major contributor to tear MMPs.

The eyelid meibomian glands collected from unwounded animals demonstrated relatively high MMP-2 and MMP-9 productions in the acinar cells. However, the current study showed little change of MMP immunoreactivity in meibomian glands after epithelial wounding and, therefore, a contribution of this glandular tissue towards tear-derived MMPs with wounding was considered unlikely.

The inner conjunctival lining of the eyelids were also explored for additional sources capable of producing MMPs following wounding. We found that CALT was associated with weak MMP-2 and strong MMP-9 immunoreactivity. This outcome is consistent with lymphoid tissue structures in the colon (Peyer’s patches), which have also been observed to express MMP-9 [40]. As such, CALT, which can easily release proteins into tears due to its close proximity with the tear film [41], [42] may be another major source of tear MMPs.

A further important contributor towards MMP levels, not investigated here, may be the polymorphonuclear leukocytes (neutrophils, PMNs) which store MMP-9 in their granules [43]. PMN cells are closely associated with corneal wound healing and can be found in both tears and the ocular surface tissue [44].

The inclusion of procedural control eyes showed that analgesic and anesthetic medication, topical eye drops and use of eyelid speculum or forceps to adjust the eyeball position during epithelial wounding caused an increase in tear MMP activity. It is hard to identify the individual contribution of each potential procedural stimulant that resulted in increased MMP expression, as their individual contributions were not tested and the outcome is likely to be a combination of several of these factors. Furthermore, the current study emphasizes the importance of using appropriate experimental controls for the analysis of tears in wound healing studies. Without using a procedural control, the analysis of tears in this study would have led to the misconception that the migrating corneal epithelium secreted most of MMP-2 and MMP-9 into tears. Future studies that employ any sort of surgery or drug treatment should also consider the use of the most appropriate control taking these factors into account.

A feline model was used in the current study to investigate the expression of MMPs in tears and the ocular surface system. Previously, the feline cornea has been used as a model for the human cornea in investigations of corneal wound healing [21]–[26], corneal implants [45], and contact lens studies [46]. The feline model, as with any other animal model, has some differences to humans including the presence of nictitating membranes and the general dimensions and size of the eye and surrounding organs. Comparison of MMP-9 concentrations to those reported for humans showed differences (slightly less MMP-9 in cats without nictitating membranes and more in cats with membranes compared to humans) [31]. These differences may be related to the nature of the feline tear film, such as different lipid (meibum) composition resulting in lower spontaneous eye blink rates, and the presence of nictitating membranes which are rudimentary in humans. Nevertheless, the feline model provided valuable insight into the expression sites of MMP-2 and MMP-9 in response to corneal epithelial wounding.

In summary, the major contributors to MMP-2 and MMP-9 found in feline tears in response to corneal wounding appear to be the lacrimal gland and CALT. Other contributors to MMP activity in tears are likely to be the corneal epithelium, stromal keratocytes and conjunctival epithelium including goblet cells. The meibomian glands do not appear to contribute at all to tear-derived MMPs after corneal wounding.

This finding may have clinical importance for various ocular surface conditions, including corneal wound healing and dry eye. The inhibition of MMP-9 in tears has been shown to promote healing and reduce inflammatory levels [47]–[49]. As the current study exemplified, the lacrimal gland and CALT are major sources for tear MMPs following wounding. It may therefore be more effective to administer MMP inhibiting medication orally rather than topically to target MMPs in these ocular surface tissues [50], [51].
